# Performance of Femininity as the Potential Determinant of Lower Well-Being Among Adolescent Girls in London, UK: An Exploratory Discourse Analysis

**DOI:** 10.1177/10497323251324385

**Published:** 2025-04-03

**Authors:** Imogen I. Hensler, Emily H. Emmott

**Affiliations:** 1 4919 University College London, London, UK; 2 4617 Queen Mary University, London, UK

**Keywords:** adolescent mental health and illness, gender, medical anthropology, anxiety, adolescents

## Abstract

In the UK, girls are consistently found to have lower subjective well-being and higher rates of anxiety disorders/depression compared to boys. While the reasons for these gender disparities are complex, how girls conceptualize, experience, and “perform” femininity may be one pathway which exacerbates psychological stress. To explore this hypothesis, we conducted an in-depth exploratory study to examine how norms and experiences of femininity among seven adolescent girls aged 16–17 from London, England (external factors), relate to their behaviors and psychological experiences (internal factors). To do so, we conducted two online focus groups in July 2021, and conducted discourse analysis to explore their conceptualizations of femininity and its impact on participants. We identified two key discourses relating to the concepts and experiences around femininity among these girls: “Valued by Conformity to Femininity,” or how girls are judged by others based on their presentation and performance of femininity, and “An Uncertain Perception of Self,” relating to uncertain self-identity stemming from their performance of femininity. We reveal a potential social conditioning process of “performing femininity” experienced by our participants, leading to hypervigilance, anxiety, uncertainty, and confusion. Our results suggest that the paradoxical and conflicting expectations around femininity, reinforced by others, may cause cognitive distortions and dissonance, increasing vulnerabilities to low well-being and anxious cognition patterns. Thus, we believe that there are grounds for further research on a larger scale which explores whether there is a sociological mechanism which is creating the gender gap in mental health outcomes at adolescence in the UK.

## Introduction

Adolescence is a sensitive period for brain development and a time when some mental health conditions can begin to emerge (Blakemore, 2019; Knopf et al., 2008; Sawyer et al., 2018). In many Western countries, studies have shown that girls’ subjective well-being often declines during early adolescence in comparison to boys, along with increased risks in internalizing behaviors and affective mental health disorders (e.g., anxiety disorders, and depression) (González-Carrasco et al., 2017; Nolen-Hoeksema & Hilt, 2009; The Children’s Society, 2020, p. 28f, 2023). While some studies show that adolescent girls and women may be more vulnerable to certain stressors, physiological differences such as earlier pubertal onset do not fully explain these sex differences, meaning other socio-cultural factors are likely at play (Natsuaki et al., 2015; Nolen- Hoeksema, 2001).

In a recent cross-national investigation, Campbell et al. (2021) found that the adolescent gender difference in mental health is found cross-culturally, with girls having worse mental health on average. Interestingly, they noted that more gender-equal countries (measured via the Gender Gap Index) have larger gender gaps across all mental health outcomes, and this has been noted as the “Equality Paradox” (Guo et al., 2024). While the causes of these findings are still unclear, it is notable that there is a positive correlation between country-level gender equality and male subjective well-being, but not female, suggesting gender-specific causal pathways (Guo et al., 2024). In the recent Global Gender Gap Report (World Economic Forum, 2023), the UK scored 0.792, making it the 15th best country in the world for gender equality (out of 146). Thus, according to these findings, the UK may be vulnerable to a comparatively larger gender gap in the mental health outcomes. Indeed, poor female mental health and subjective well-being among children and adolescents in the UK, comparatively to males, has been a consistent finding for almost a decade (Girl Guiding, 2021; The Children’s Society, 2023; WHO, 2017). Yet, the socio-cultural mechanisms which may lead to this observed gender inequality have been under-studied. Thus, in this paper, we explore why UK girls are at greater risk of poor mental health and subjective well-being, and in particular what socio-cultural factors could be contributing to this.

### Self-Construct and Well-Being

Several studies have shown links between identity-related constructs and affective disorders during adolescence. For example, Gittins and Hunt (2020) found that self-esteem is closely connected to depressive symptoms through a reciprocal relationship, meaning low self-esteem may trigger or worsen depressive symptoms. Studies also show that adolescents in a state of ambiguity regarding their self-identity are more likely to experience poorer mental health outcomes, including socio-emotional problems (Meeus et al., 2012; Potterton et al., 2022). In addition, research has shown that adolescent boys are more likely to be self-assured and confident than girls (Cole et al., 1999; Dahlbom & Kotsadam, 2011; Luscombe & Riley, 2001), which may explain why girls tend to experience lower levels of well-being compared to boys at pubertal onset (Blakemore, 2019; Sawyer et al., 2018). While the causes behind the sex differences in self-concept are unclear, it may be possible that the socio-cultural environments in countries such as the UK encourage conflicting self-concepts leading to poorer mental health outcomes for girls.

Notably, adolescence is often a period of gender intensification, where gender roles are reinforced via gender-differentiated socialization (Hill & Lynch, 1983). In contemporary Western societies, adolescents experiment with gender performance and identity (Butler, 1988; Steensma et al., 2013) where they present themselves (dressing, acting, and behaving) based on a mixture of masculine and feminine norms. While femininity is not limited to women (Hopkins & Richardson, 2021), femininity is often seen as an expression of female-typical gender (Cameron, 2005). Thus, for many adolescent girls, femininity is likely to be an important component of gender intensification and performance. Hill and Lynch (1983) noted that gender intensification can coincide with the onset of mental health problems and hypothesized that the pressures on adolescent girls to conform to culturally sanctioned gender roles may lead to stress, leading to a higher risk of depression. Similarly, Natsuaki et al. (2015) proposed that a feminine gender-role identity may increase internalizing psychopathology (e.g., anxiety disorders and depression) due to pubertal timing and gender-role identity. They state that these factors interact alongside one another such that a risk in one factor can be triggered (or enhanced) by a strength in another factor. However, to date, studies on the relationships between concepts of gender, self-identity, and mental health during adolescence are limited. In the available literature, one longitudinal study of 410 adolescents in the US found that adolescents with more feminine gender-role identities were associated with greater depression symptoms (Priess et al., 2009), although it is not clear why such an association exists.

Overall, while it has been long hypothesized that gender constructs and conflicting identity may cause girls to be more vulnerable to low subjective well-being and affective disorders, we are yet to fully understand the basics of how gender is conceptualized by adolescent girls in the UK, how it is performed, and how this may relate to their experiences of stress.

Thus, to understand the female adolescent experience, we broadly investigated the concepts and experiences of femininity via small and in-depth online focus groups. Taking adolescence to be a period of gendered identity development, exploring the cultural concepts and performances of gender in relation to self-identity may reveal the mechanisms behind why girls are comparatively vulnerable to lower well-being in the UK. In this paper, we aim to contribute to this knowledge by documenting how an ethnically diverse group of adolescent girls from London think about femininity and their related experiences. In turn, this may shed light on the potential socio-cultural mechanism behind low subjective well-being and the mental health of adolescent girls.

### Aims and Objectives

In this study, we investigate (1) the concepts and experiences of femininity among adolescent girls and (2) if and how these concepts and experiences impact girls, with specific focus on their subjective well-being. By conducting discourse analysis on focus group data involving seven adolescent girls aged 16–17 years from London, we provide an in-depth investigation into the views and lived experiences of femininity among this group of young people. In doing so, we reveal the potential processes and mechanisms of how conceptualizations of contemporary femininity in London may impact adolescent well-being.

## Methods

### Recruitment and Participants

We recruited seven adolescent girls between 16 and 18 years old (mean age = 16.4) from North and East London, England. This location was chosen for several reasons, including convenience, cultural diversity (to gain understandings of femininity which reflected London), and the authors’ in-depth knowledge of the local socio-cultural context which is integral to discourse analysis. Participants were recruited via posters in youth centers and word of mouth, including snowball sampling (i.e., recruiting the participant’s friend). All participants gave written and verbal informed consent to take part, and were given a £5 Amazon gift voucher and a certificate of participation after the online focus group as a token of thanks. Caregiver consent was not required as participants were 16–18 years old, but we encouraged participants to discuss participation with relevant caregivers. Participant details for each focus group are outlined in Table 1.

**Table 1.** Participant Information and Focus Group Allocation.

Group	Name (Pseudonymized)	Place of Residence	Age	Self-Described Ethnicity*
Focus Group 1	Aaliyah	North London	16	Congolese
	Eloise	North London	16	British
	Josephine	North London	16	Black British
	Sara	North London	16	Middle Eastern
Focus Group 2	Emily	Greater London	17	White
	Holly	Greater London	17	White
	Suzanna	Greater London	17	White/Asian

Shown are the participant (pseudonymised) information and group allocation. *Participants were asked for a “self-description” of ethnicity in order to not give prescriptive categories of ethnicity.

### Data Collection

The study used focus groups to capture open communication and gain individual and group-level understandings of femininity. This suited our project aims of exploring how adolescents conceptualize femininity, as focus groups demonstrate group understandings and generalized thinking (Kitzinger, 1994) while facilitating open communication (Powell & Single, 1996).

We ran two online focus groups lasting 1.5 hours each, with 3 or 4 participants in each group. The focus groups took place in June/July 2021 via Microsoft Teams, and were facilitated by the lead author. All participants and the lead author had their cameras on during the focus group, and the session was recorded directly using Microsoft Teams. The participants within each focus group knew each other/were friends, but not between focus groups.

The focus groups were conducted in a semi-structured manner, with pre-planned open questions as well as spontaneous follow-up questions by the main researcher. To facilitate discussion, the focus groups included icebreakers, anonymous polling via slido.com (an online tool to allow real-time Q&As/surveys), as well as group discussion with further prompting questions. Interactive anonymous polling was specifically used to “ease in” participants and promote engagement, as anonymous ways to respond as this can alleviate stress and lead to more truthful answers (Ong & Weiss, 2000). Here, participants were asked to submit anonymous answers to set questions at the beginning and end of the focus group interview, which was seen and discussed by all participants, and used as topic starters by the researcher for further questions. Overall, the lead author’s impressions were that participants were open and comfortable speaking and sharing their thoughts within the focus group, as evidenced by active participation by all participants including contributing personal thoughts and experiences. Further information of the focus groups, including the topic guide, question matrix, and focus group processes can be found in the Supplemental Information.

### Analysis

Discourse analysis is commonly used in adolescent gender studies to capture social practises (Duke & Kreshel, 1998; Hauge, 2009; Leahy, 1994). Here, we conducted critical discourse analysis where we assume that “social realities […] [are] humanly produced constraints” (Fairclough, 2012, p. 10), where the language and conversations that appear in focus groups reflect gender inequalities and associated social mechanisms experienced by adolescent girls in London. We focused on identifying discourses which highlighted concepts of femininity through the interplay of social structures, practices, and events (Chouliaraki & Fairclough, 1999) which impacted the adolescent girls. One advantage of discourse analysis is that, due to the focus of understanding the processes and mechanisms behind social inequalities (rather than describing population-level patterns), the method is less impacted by sample size (Potter & Wetherell, 1987).

Each focus group was automatically transcribed full verbatim via Microsoft Teams, then the transcripts were reviewed by the lead author for word errors. Additional details were further added, such as non-verbal cues. For each focus group, final transcripts were inductively analyzed by the lead author with particular focus on linguistic content and the socio-cultural context to identify key discourses. The emerging discourses were then cross-referenced between the focus groups, which initially led to four main discourses (see Table 2 in the Supplemental Information to review all the discourses). To improve the validity and reliability of the findings, the second author read extracts of the transcripts and discussed the emerging results and discourses with the lead author. Of these final four discourses, two directly aligned with the current research question. Below, we outline the two relevant discourses: *Valued by Conformity to Femininity* and *An Uncertain Perception of Self*.

## Results

### Discourse 1: Valued by Conformity to Femininity

I don’t exactly have to be feminine, but I feel like in order to be accepted I still have to. (Aaliyah, 16, Focus Group 1)

All participants in our focus groups discussed how they were monitored and judged by others based on their femininity, such as being passive/submissive. Both groups acknowledged that acting feminine makes them accepted and liked by others, specifically their peers, and can also diffuse challenging social situations. This led participants to modify their behaviors in an effort to increase their social standing and/or as protection.

### Consequences of Femininity

All participants raised that their everyday behaviors led to social consequences based on how they perform and conform to femininity. The girls noted that when they or others perform in a conventionally feminine manner, they receive social benefits—particularly from boys and men. For example, this was explicitly noted by Sara in Focus Group 1:

**Sara:** She also has to be attractive and kind of like feminine to a certain point. Uh, and that’s still appealing to men, in order to kind of gain their validation or like acceptance and society, you kind of do have to be a level of feminine.

In direct contrast, deviating from stereotypically female behaviors and personas often led to negative social interactions. For example, participants expressed that sharing opinions confidently will come across as “argumentative,” and consequently they will be branded as “difficult,” as exemplified by the conversation in Focus Group 2:

**Emily:** When you debate with the boys, you often labelled as like, oh like [inaudible], but if the boys were to like, say the same thing and whatever, it's just like, oh, they’re just saying their opinion. If it’s the girls like really going after her opinions, it's like wow like look at her go. I feel like it is kind of different.

--

**Suzanna:** [My friends and I] will spend a lot of lunchtimes talking about, like issues, and then we always hear from other people that like the boy group sat near us have heard and now they’re angry with us because they think our ideas are like not right or something. There are quite a lot of issues with that.

--

**Holly:** Going into the conversation I can be argumentative, but then, like by the end like I’ll have to be like laughing it off to sort of avoid it going any further.

Emily’s statement suggests that the lack of conformity to the feminine stereotypes of “softness” and “passiveness” during conversations, such as sharing opinions confidently, can lead to negative labels—suggesting girls are often bound to performing femininity and are judged to these standards, while boys are not. Suzanna’s example shows how negative consequences of “not being the right kind of feminine” can occur even when boys are not directly involved in the conversation. This may reflect the external surveillance and monitoring girls experience, and how others may feel entitled to pressure girls to modify their thoughts and behaviors. Holly’s statement exemplifies how, even in situations where girls enter a conversation with confidence, they hold the burden of appeasement leading to their performance of submission.

### Performing Femininity for Benefits and Protection

Throughout both focus groups, the girls expressed how they actively modify their behavior “to be more feminine” in order to mitigate against negative social interactions. For example, the girls change the way they discuss topics and/or become passive in conversations, particularly with male presence, because not behaving in this way could lead to trouble. Participants discussed that they may “dumb themselves down” in order to make themselves “more appealing to men.” When asked how she would make herself more appealing to men, Eloise from Focus Group 1 stated that it could be in numerous ways:

**Eloise:** It could be [making yourself more] attractive, or it could be like making it sound like an ego boost for them. If you’re like weak, you can be like the damsel in distress and they can, you know, have a bit of like an ego boost out of that.

Interestingly, Eloise, who self-described as being very feminine since she was a young girl, explicitly referred to her femininity as a “tool.” However, she also stated that when she realized that this tool is a product of the male gaze, it left her feeling “disillusioned”—suggesting that using femininity as a tool is not necessarily an empowering experience but a strategic method to navigate the socio-cultural constraints placed around girls. Both groups referred to how performing femininity can be a confusing interplay between using their femininity to their advantage, but realizing this performance is to appease and pacify others. When Focus Group 2 was asked how the standards and expectations of femininity impacted them, they did not explicitly answer the question, but they did raise an intriguing point–that engaging with their feminine side is “safe”:

**Suzanna:** I think sometimes it feels almost like safer to do it. Like sometimes I would like oh I can’t be bothered to wear makeup today to school and then I’m like- oh no, but then I’ll look weird and they will think oh she looks so ugly. So then I just do it anyway ‘cause it just feels like the safer option. And it’s just easier at that point.

**Holly:** Yeah.

**Suzanna:** […] Some days I’m like oh I can’t be bothered and then I go I’m thinking like I’m only doing this so that like other people won’t look at me strangely.

**Emily:** Yeah. 100%. And just like I, I might regret it if I don’t. But yeah, it’s just about. I feel like safety and I guess that makes you comfortable. But if it weren't for other people, then I probably wouldn’t.

**Suzanna:** Yeah, it is easier to just conform sometimes.

When asked what they meant by “safe,” they stated that it was safety from the scrutiny of others and getting negative attention by peers. This reflects how performance of femininity can be a pre-emptive/defensive safety behavior. However, these safety behaviors such as makeup wearing are not necessarily empowered actions. As Suzanna’s statement below exemplifies, girls can feel forced to meet certain standards of femininity to even be considered and valued as a person:

**Suzanna:** I agree as well that like you kind of have to like. Put on like an act. If you when you first meet someone new like a new boy because it’s like. Like it’s almost like if you don’t meet what they expect, they won’t even give you the time of day to like become friends or anything. So it’s like you kind of have to like. It’s not really like an option.

### Discourse 2: An Uncertain Perception of Self

Every time I make a decision, I think am I doing this for me or am I trying to appeal to them. (Sara, 16, Focus Group 1)

Overall, participants seemed to experience uncertainty over themselves stemming from how they were externally judged and valued based on their conformity to femininity. The social judgments they experienced meant participants constantly attempted to meet the expected standards of femininity, leading to detailed and meticulous analysis of how to present themselves. These patterns of thoughts mirrored hypervigilance, overthinking how different behaviors and actions could lead to different social consequences. At the same time, they also believed that these standards of femininity are problematic in and of themselves, with doubts around the effort they expended to perform them. Consequently, girls had underlying questions and self-doubt around their identity.

### Hypervigilance

Hypervigilance refers to an acute awareness of your surroundings, how you are being perceived, and how you are presenting yourself. Both focus groups noted that they are constantly aware—or hypervigilant—of being judged by others, because they have directly experienced or witnessed it first-hand. Focus Group 2 shared several experiences relating to this issue:

**Holly:** When I’m with [my male friends] they’ll be like criticizing other girls’ appearances and like calling her ugly and stuff and it’s like now I know you’re saying that type of thing about me behind my back like they very clearly do care about looks and they do put emphasis on that.

[…]

**Holly:** That’s the thing, like [boys] literally do [criticize and judge girls on their appearance]. Like [my friends and I] were at Reading Festival and like it happened to one of my friends. Like some guy was like, oh no she’s so ugly, to this girl like literally right to her face […] it’s not just something that goes on in our heads like it actually is a thing.

--

**Suzanna:** […] But then like every time I talk to a male friend, or like a boy in the year or something like it comes to mind a lot more and you’re thinking a lot more about like. Like how are they present like how are they viewing me right now and stuff like that.

--

**Sara:** […] I don’t know how to say this, but. ‘cause I think male validation has become a big… It has always been a big thing, in femininity. (*Eloise nods in agreement*)

These conversations reflect how acutely participants are judged on their external appearance, which was a particular concern for participants in Focus Group 2. For both focus groups, the judgments and the negative consequences participants experienced framed male opinion as highly important, and indeed, both focus groups raised how they were sensitive to boys’ opinions. As Suzanna’s statement above exemplifies, because of these experiences, girls paid significant attention to the judgments being made by boys. These interactions and behaviors could lead to low confidence and worries:

**Suzanna:** I have quite normal self-confidence and then as soon as I start talking to someone like a boy that I know will say negative stuff, I start thinking like oh my skin’s horrible today, like actually scrutinizing myself as well, thinking about my insecurities.

Interestingly, self-monitoring could also be externally performed to navigate complex expectations around “appropriate femininity” and as a form of protection:

**Holly:** I’ll say like oh like I look bad today like ha ha. To stop other people being like - oh she’s got so much confidence, who does she think she is? She’s so ugly, like you know. ‘Cause you just want to be the person to be like - oh, I know, you don’t need to say it.

Holly’s statement highlights how girls are judged on their appearance with pressures to conform to particular standards of beauty. Yet, doing so confidently conflicts with the expectations around femininity to be passive and submissive. Pre-emptive self-depreciation is therefore used as tool to mitigate negative social implications. This reflects the highly complex performance of femininity our participants navigate, with pressures to balance the different domains of femininity (appearance, personality) which requires significant focus on both internal and external judgments about being feminine. Both focus groups raised that they feel as though they are under surveillance, being continuously judged for “the little things.” Arguably, this level of monitoring, hypervigilance, and pressure is exhausting for participants. However, participants felt unable to “reject” these pressures, as exemplified by Eloise in Focus Group 1:

**Eloise:** Yeah, I agree with that and I think it is like quite hard feeling that you are being watched and judged all the time because. It’s sort of like it's a lot of pressure. We sort of. We want to. Reject these things and not care, but we’ve got this pressure coming from like outside like even just leaving the house you want. You’re thinking like, oh, do I look OK? And it’s like it doesn’t really matter. ‘cause you know if you’ve got things to do like that’s all that should matter. You're going to do them. Why does it matter what you look like? But because you’re constantly feeling like the pressure from society and feeling that you’re being watched? It’s sort of hard to … reject that.

### Confusion

Eloise’s statement above also reflects an internal conflict whereby there is conscious acknowledgement that making yourself conventionally look attractive, “should not matter,” yet they continue to perform to meet this standard of beauty. In both focus groups, participants discussed the dilemmas between accepting versus rejecting femininity and presenting as more versus less feminine. This led to self-doubt and ambiguity around their identity where “you don’t know who you are,” which was raised explicitly by participants in Focus Group 1:

**Sara:** I think it definitely kind of impacts you in that. You’re not you kind of. You don’t know how you want others to see you because if you’re only feminine that can be seen as a bad thing. But if you completely reject it, I don’t know, I feel like all in all it’s just all like kind of being submissive to the male gaze, it feels like you can never win. So that can kind of create like an identity crisis. Like we said earlier where you kind of don’t know who you are and what you want to be seen as.

--

**Sara:** Yeah, I think it. It kind of confused me like every time I make a decision now I’m kind of thinking at the back of my head. Oh, am I doing this for me or am I doing this for you know to try to appeal to them in some way? Yeah.

--

**Josephine:** I feel like sometimes it’s hard to act your actual self and you don’t really know how you normally would act around people that aren’t male.

**Eloise:** Yeah, it’s kind of difficult to like know your own identity in a way ‘cause there’s like the idea of what you think you are, but then that’s obviously with all the societal factors and what everyone is trying to tell you to be like as a woman. So, if you took away all those factors, it’s hard to know what you would actually be like, and then it’s bit of like an identity crisis (*Eloise laughs slightly*).

Participants in both focus groups mentioned that they wish to reject these societal standards and messages of femininity, yet the pressures to perform these standards of femininity is pervasive through their lives, coming from parents and peers as well as wider society. This led to an internal battle around self-identity, trying to fathom how they wish to be, while constantly being aware of how they are being externally judged/perceived and the social risks that come with it. “Becoming yourself” was therefore highly constrained within “being feminine,” which, consciously, they wished to reject. This led to high levels of worry and uncertainty.

## Discussion

Overall, the participants reported being persistently judged based on their conformity to femininity, particularly by male peers at school. For example, participants discussed how social interactions are often more positive when they make themselves more “palatable” and present as more stereotypically attractive. This lived experience directly conflicted with their own beliefs around how girls and women should be treated and valued by peers and society, leading to high levels of uncertainty in self-concept. Similar paradoxical experiences have been raised repeatedly in conjunction with the “impossible standards” of being a woman in English society (Bates, 2016).

**Figure 1.**
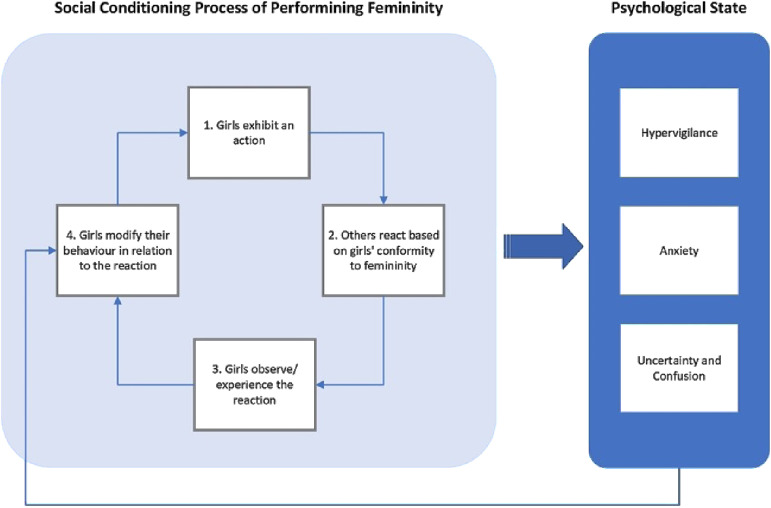
Social conditioning process of performing femininity and the subsequent psychological states.

Importantly, the results as outlined in *Valued by Conformity to Femininity* point to a social conditioning process whereby (1) girls exhibit an action, (2) others (primarily male peers) react to this action based on the girls’ perceived conformity to femininity, (3) girls observe/experience this reaction, and (4) modify their behavior in relation to this reaction (Figure 1). This reflects how girls may be inadvertently encouraged to perform to certain stereotyped forms of femininity, sometimes for their own protection. As outlined in *An Uncertain Perception of Self*, this leads to hypervigilance surrounding their own behavior, appearance, and the social judgments being made. The process also situates the girls as being primarily responsible for the outcomes of social interactions, and they experience the burden of modifying their behavior. The fault of any negative interactions is therefore attributed to the girls, which may lead to anxiety, worry, and lower self-esteem. To mitigate these risks, girls engaged in “behaviors of safety,” such as fixing their hair and wearing makeup. Note, these behaviors directly mirror items in the Safety Behaviour Questionnaire (e.g., “Wear clothes or makeup to hide blushing”; SBQ; Clark, 2005), which is a self-report questionnaire that measures the frequency of safety behavior usage in the context of anxiety symptoms. Increased SBQ scores correlate with social anxiety symptoms in both community and clinical samples (Evans et al., 2021).

The gender disparity in subjective well-being and mental health among UK adolescents is notable, with the estimated prevalence of anxiety disorders being 10.3% for boys and 23.9% for girls aged 17–20 years (Early Intervention Foundation, 2020). Our findings suggest that performance of femininity and the pressures to do so may be contributing toward lower subjective well-being and poorer mental health among girls in the United Kingdom. Specifically, we hypothesize that the social conditioning process identified below may encourage cognitive distortions among girls, such as externalization of self-worth, co-occurring with hypervigilance, excessive attention to mind-reading, and low self-esteem (Albano & Barlow, 1996; Beck, 1963; Widdowson, 2014; Yurica & DiTomasso, 2005). Hypervigilance is commonly linked to anxiety disorders due to the emphasized awareness of an individual’s surroundings and scanning for threats (Richards et al., 2014), while low self-esteem has been associated with lower subjective well-being and poorer mental health (Diener, 2009; Silverstone & Salsali, 2003; Sowislo et al., 2013).

Additionally, the social conditioning process experienced by the girls may create cognitive dissonance, as it directly contradicts their own beliefs around how girls and women should be valued and treated by peers and society. This is likely to amplify any uncertainty in self-concept, which is associated with lower subjective well-being and poorer mental health (Gittins & Hunt, 2020; Meeus et al., 2012; Potterton et al., 2022; Suh, 2002). While some scholars have argued that adolescence is a time of identity development with uncertainty being inherent to the process (Crocetti, 2017; Erikson, 1968; Klimstra & van Doeselaar, 2017), anthropological studies have long evidenced that this is not universally experienced across cultures (Hewlett & Hewlett, 2012; Mead, 1928), suggesting socio-cultural factors may be at play. Our results indicate that the social and cultural environment experienced by adolescent girls in the United Kingdom may place them at particular risk of acute levels of uncertainty and related anxiety.

It is interesting to note that girls reported the use of “safety behaviors” (Salkovskis, 1991) to mitigate/navigate potentially stressful social interactions and situations. While such behaviors can facilitate the engagement with an anxious event, they can also perpetuate anxiety in the long term, and such behaviors are associated with various mental health disorders (Morgan & Raffle, 1999; Salkovskis, 1991; Simpson et al., 2012), and particularly to anxiety disorders (Clark, 1999).“Impression-management” safety behaviors are those which aim to produce a good impression socially such as being fixated on how they may be perceived, and these behaviors are more commonly used by older adolescents (16–18 years old) (Evans et al., 2021). In the present study, the participants exhibit a social conditioning process which is arguably catalyzed by these “impression-management” safety behaviors such as fixating on how they are coming across in conversations and adapting behavior to create better social outcomes.

To date, studies examining the gender gap in mental health have tended to focus on low-mood, depressive symptoms, and low self-esteem, and less so on anxious symptoms and behaviors (Hyde & Mezulis, 2020; Piccinelli & Wilkinson, 2000; Van de Velde et al., 2010). We hypothesize that adolescent girls in the United Kingdom may be at particular risk of elevated levels of anxiety due to the high-risk nature of social interactions, whereby the outcomes are underpinned by the girls’ performance of femininity. Thus, concepts of femininity may be contributing to the onset of anxiety disorders at adolescence.

### Limitations

Our findings are based on a small sample of adolescent girls aged 16–17 living in London, England, and their experiences are highly influenced by their local socio-ecological context. This means we must be particularly cautious about extrapolating results to other groups of adolescent girls. From the current findings, we are not able to infer whether the described social conditioning process relating to concepts of femininity is experienced across other parts of England or the United Kingdom. Instead, our findings inform a hypothesis surrounding potential mechanisms which may explain, at least in part, why adolescent girls in the United Kingdom are at higher risk of lower subjective well-being and poorer mental health. Further, this study employed an online interactive focus group as a data collection method. This was primarily due to social distancing restrictions imposed during the COVID-19 pandemic. It is not clear whether our current findings have been impacted by an online method, although participants were clearly familiar with online group video calls, and participants seemed comfortable sharing their thoughts and experiences with the researcher as indicated by sharing of personal experiences.

### Future Research Implications

This study’s rich qualitative data is a great resource to understand that conceptions of femininity, in relation to adolescent mental health, are worth investigating further and on a larger scale. One reason that Campbell et al. suggested that more gender-equal countries have larger gaps in mental health is because there are confusing and expansive gender standards (Campbell et al., 2021, p. 7) which was reflected in the results from this study. With adolescent girls continually reporting poorer mental health and well-being than adolescent boys in the United Kingdom, an important sociological mechanism could be discovered.

### Positionality Statement

The lead author is a white, middle-class woman who grew up in England and, under some definitions, would have been classed as an adolescent at the time of data collection. The lead author’s relatively recent experiences of secondary schooling promoted a deeper understanding of the nuances around participants’ experiences of femininity. This shared background enabled a unique insider perspective, enriching the interpretation of findings with in-depth comprehension of the social and cultural contexts influencing adolescent girls’ performances of femininity.

However, this shared identity and experience also introduced the potential for bias, as the lead author’s own perceptions and experiences of femininity could unconsciously shape interpretations of the data. Recognizing this, measures were taken to mitigate such biases, including reflexive practices and engaging in dialogues with the second author to challenge and broaden the lead author’s perspectives. In particular, the second author, being in the 30s, bi-racial, and having grown up in bi-cultural contexts, brought a diverse set of experiences and viewpoints that were instrumental in providing objectivity and depth to the analysis. This approach aimed to ensure that the analysis remained grounded in the participants’ experiences rather than being overly influenced by the lead researcher’s personal journey.

## Supplemental Material

Supplemental Material - Performance of Femininity as the Potential Determinant of Lower Well-Being Among Adolescent Girls in London, UK: An Exploratory Discourse Analysis Supplemental Material for Performance of Femininity as the Potential Determinant of Lower Well-Being Among Adolescent Girls in London, UK: An Exploratory Discourse Analysis by Imogen I. Hensler and Emily H. Emmott in Qualitative Health Research
