# Supplementary material for: Performance of Femininity as the Potential Determinant of Lower Well-Being Among Adolescent Girls in London, UK: An Exploratory Discourse Analysis
Source: Qual Health Res. 2025 Apr 3;36(7):658–68. doi: 10.1177/10497323251324385 (PMC13135648; doi:10.1177/10497323251324385)
Supplement: Supplemental Material - Performance of Femininity as the Potential Determinant of Lower Well-Being Among Adolescent Girls in London, UK: An Exploratory Discourse Analysis [file sj-pdf-1-qhr-10.1177_10497323251324385.pdf]

Supplementary Information

Contents

Supplementary Information 1
Ethics Approval Reference Numbers 1
Data Collection: Focus Group Details 2
Focus Group Topic Guide 2
Focus Group Question Matrix 3
Focus Group Planned Process 3
Data Analysis 6
Coding Transcripts 6
Process for Identifying Discourses 6
Process for Finalising Discourses 7
Process for Increasing Reliability of Discourses 8

Ethics Approval Reference Numbers

1. UCL REC Ethics (number: 20487/001)
2. Data protection (number: Z6364106/2021/03/165)
3. Enhanced DBS (Certification number: 001736081787)

Data Collection: Focus Group Details

The lead author (Hensler) conducted the focus group under the guidance of Emmott.

Focus Group Topic Guide

1. When I say “femininity” what words does it immediately make you think of?
    a. [slido Q; word cloud] → then ask the group about these specific words
2. On a scale of 1-10 how feminine would you say you are?
    a. [slido Q]
3. If a woman was very feminine what would she look like?
4. If a woman was very feminine what would she act like?
5. If a man was very feminine what would he look like?
6. If a man was very feminine what would he act like?
7. The Oxford dictionary defines femininity as “qualities or attributes regarded as characteristic of women”. Do you agree with this?
8. What would be your definition of femininity?
9. What is your relationship with your feminine side?
    a. Do you accept your femininity or do you reject it?
10. Do you feel like you have to be feminine?
    a. Why?
11. Do you find it easy or hard trying to navigate these gender dynamics within yourself?
12. Is there an impact through engaging with your feminine side?
13. On a slightly different note - What it's like being a teenager in UK today
    a. [slido Q]

Focus Group Questions

	Question	Research Question it Relates To
1.	When I say “femininity” what words does it immediately make you think of? [slido Q; word cloud] → then ask the group about these specific words	1
2.	On a scale of 1-10 how feminine would you say you are? [slido Q]	1
3.	If a woman was very feminine what would she look like?	1
4.	If a woman was very feminine what would she act like?	1
5.	If a man was very feminine what would he look like?	1
6.	If a man was very feminine what would he act like?	1
7.	The Oxford dictionary defines femininity as “qualities or attributes regarded as characteristic of women”. Do you agree with this?	1
8.	What would be your definition of femininity?	1
9.	What is your relationship with your feminine side?	2

	Do you accept your femininity or do you reject it?	
10.	Do you feel like you have to be feminine?<br>Why?	2
11.	Do you find it easy or hard trying to navigate these gender dynamics within yourself?	2
12.	Is there an impact through engaging with your feminine side?	2
13.	On a slightly different note - What it's like being a teenager in UK today<br>[slido Q]	2

Table 2: Focus group interview question matrix. Note, 1 = the concepts and experiences of femininity among adolescent girls; 2 = if and how these concepts and experiences impact girls, with specific focus on their subjective-wellbeing.

Focus Group Planned Process

1. Participants log onto the Microsoft Teams link
2. Participants are admitted once there are multiple participants in the ‘lobby’.
3. Participants join with video and audio. Once all the participants are in the call, the recording will begin.
4. Participants are read out an overview of the study and be able to ask any last minute questions.
5. The group will be asked a set of opening questions and participants will reply anonymously using slido.com using a mobile phone/tablet.

6. These answers will be used as topic starters, where I will ask further questions based on these responses.
7. The interview will then follow in this casual format whereby participants will be asked a question as a group and participants will answer when you desire.
8. Once I have covered all the appropriate topics, participants will be informed that the interview is over.
9. Participants will receive a debrief and a reminder of the contact details and aims of the study.
10. The recording will then stop and the interview will be over.
11. The call will then end.

Data Analysis

The lead author (Hensler) was the primary researcher for the analysis, under Emmott's guidance and input who also contributed in the later stages of analysis.

Coding Transcripts

1. FG1 was read through and themes were mentally noted
2. Hensler decided on 4 main themes
    a. stereotypical notions of gender
    b. comments of strength in women
    c. societal notions
    d. personal opinion
3. Hensler highlighted participant quotes that demonstrated a theme and annotated with further comments to aid analysis.
4. FG2 was read through and themes were mentally noted
5. Hensler decided on 4 main themes
    a. stereotypical notions of gender
    b. comments of strength in women
    c. societal notions
    d. personal opinion
6. Hensler highlighted quotes by participants which demonstrated a theme and annotated with further comments to aid analysis.

Process for Identifying Discourses

1. Hensler complied all the discourses that were relevant from the FG1 annotations on a document.

2. Hensler then re-read FG1 and noted every time there was a piece of evidence of a discourse.
3. Hensler then looked at how prevalent each discourse was and finalised it to 4 main discourses of FG1.
4. Hensler compiled all the discourses that were relevant from the FG2 annotations on a document.
5. Hensler then re-read and noted every time there was a piece of evidence of a discourse.
6. Hensler then looked at how prevalent each discourse was and finalised it to 4 main discourses FG2.

Process for Finalising Discourses

1. BG's main discourses were compared to notice any obvious discourse overlap
2. Hensler then went through both transcripts page by page in conjunction with each other and wrote all the discourses apparent in both and how many times they came up
3. Hensler then finalised the 4 most prominent discourses from both
    a. Everything is for him
    b. Relationship with femininity changes with age
    c. Uncertain perception of self
    d. Judged based on conformity to femininity
4. Hensler then made unannotated versions of each focus group's transcript
5. Hensler then re-read FG1's and highlighted quotes by participants which demonstrated a discourse, and annotated with further comments to aid analysis.

6. Hensler then re-read FG2's and highlighted quotes by participants which demonstrated a discourse, and annotated with further comments to aid analysis.
7. Hensler then analysed the transcripts together and reviewed discourses by discourse and made notes on the evidence of each discourse.
    a. These notes were the main source used to write up the Results section.

Process for Increasing Reliability of Discourses

1. Once Hensler had completed the analysis and finalised discourses

2. Hensler and Emmott had a meeting to discuss the discourses, by looking through both transcripts

3. Hensler then explained how she had identified and finalised the discourses

4. We then looked over my annotated transcripts and had a discussion based on the notes Hensler had made.

5. Hensler then showed Emmott all of the discourses from each stage (initial discourses and finalised discourses)

6. Emmott then asked questions and probed why and how Hensler had concluded that these were the final discourses.

7. Emmott then outlined her opinions of the finalised discourses based on the transcripts

8. Both researchers then had a further discussion about their differing and similar opinions.

9. Both researchers then found a mid-ground and amended my discourses to fit both analyses.

**Focus Group 1 Discourses**	**Finalised Focus Group 1 Discourses**	**Focus Group 2 Discourses**	**Finalised Focus Group 2 Discourses**	**Overall Discourses**
Femininity as a tool	Femininity as a tool	Everything is for Him	Everything is for Him	Everything is for Him
Identity splitting/identity crisis		Emotional		
Femininity shapeshifting through chronological and biological age		Caring		
Judgement based on femininity conformant	Awareness and conceptions of femininity leads to identity crisis	Male validation	Opinions are not taken seriously	Relationship with femininity changes with age
A togetherness through shared experiences of negative events		Rejection of femininity when younger		
A worry of being silenced or disrespected for their thoughts		Fear of being a social outcast		
Torn between using or rejecting it	Femininity shapeshifting	People pleasing	Fear of being a social outcast	Uncertain perception of
Feeding into male expectations		High emphasis on appearance and being critiqued for it		

<table>
<tr><td>Women are allowed a more flexible spectrum of their sexuality in comparison to men</td><td>through chronological and reproductive age</td><td>Engaging with feminine side is safer and easier</td><td></td><td>self</td></tr>
<tr><td>Hard to create a definition of femininity when it is complex</td><td rowspan="3">Judged based on conformity of femininity</td><td>Opinions are not taken seriously</td><td rowspan="3">Engaging with feminine side is safer and easier</td><td rowspan="3">Judgment based on conformity to femininity</td></tr>
<tr><td>UK femininity is hard to adhere to in relation to ethnic standards of womanhood</td><td>Femininity can bring deeper and more meaningful connections with others</td></tr>
<tr><td>Being in London gives a flexibility of femininity</td><td>Selfless</td></tr>
</table>

Table .3: Discourses at each stage. Shown are the discourses from each stage of analysis. The original 4 discourses are the ‘Overall Discourses’
